# Severe antibody‐mediated transfusion‐related acute lung injury in an obstetric patient following transfusion of fresh frozen plasma from a non‐transfused male blood donor

**DOI:** 10.1002/ccr3.3818

**Published:** 2021-06-10

**Authors:** Charlotte Nielsen Agergaard, Thure Mors Haunstrup, Anne‐Louise Fjordside, John Baech, Rudi Steffensen, Kaspar René Nielsen

**Affiliations:** ^1^ Department of Clinical Immunology Odense University Hospital Odense Denmark; ^2^ Department of Clinical Microbiology Vejle Hospital University Hospital of Southern Denmark Vejle Denmark; ^3^ Department of Clinical Immunology Aalborg University Hospital Aalborg Denmark

**Keywords:** neutrophil antibodies, plasma, TRALI, Transfusion

## Abstract

Transfusion‐Related Acute Lung Injury (TRALI) has been associated with neutrophil reacting antibodies in transfused blood products. We report a case of life‐threatening TRALI in an obstetric patient triggered by transfusion from a non‐transfused male blood donor. A residual risk of TRALI exist, even in a male‐only plasma setting.

## Graphical abstract

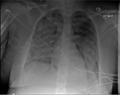

## 1 INTRODUCTION

Transfusion‐related acute lung injury (TRALI) is a clinical diagnosis defined by the Canadian Consensus Panel.^1^ The diagnostic criteria include acute onset hypoxemia during or within 6 h of transfusion, bilateral infiltrates on the chest radiograph and exclusion of other risk factors for acute lung injury (ALI).^2^ Life‐threatening cases of TRALI can be provoked by the presence of neutrophil reacting antibodies in the transfused blood product.^3^ These antibodies are often related to multiparous women; thus, most countries use male plasma only for transfusions. This practice has substantially reduced the risk of TRALI. However, a residual risk persists; therefore, transfusion therapy must be goal‐directed to diminish the risk of over‐transfusion and adverse effects.^4^ An estimate from recent hemovigilance data have reported a frequency of TRALI of approximately 1/65.000 transfusions.^5^

## 2 CASE REPORT

**FIGURE 1:**
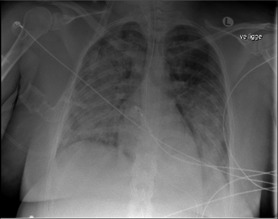
Chest X‐ray showing bilateral infiltrates

**FIGURE 2:**
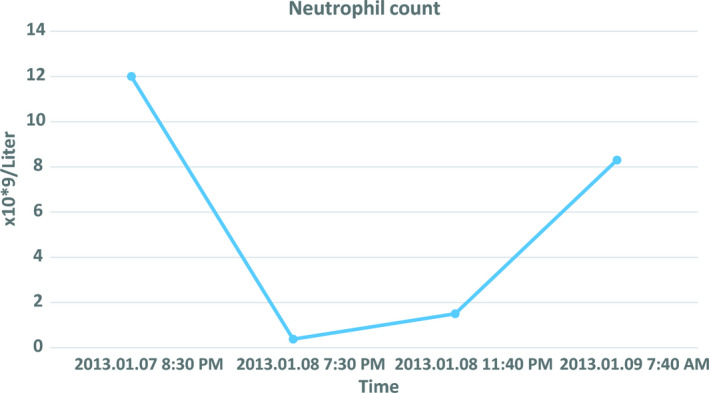
Neutrophil count showing a drop after the onset of symptoms

A 34‐year‐old nullipara woman with no past medical history was admitted to the hospital at 41 + 5 weeks of gestation. Acute cesarean delivery was performed, due to failure to progress, and a live male infant was delivered. Eight hours postpartum, the patient's condition deteriorated with severe abdominal pain, a fall in hemoglobin (12.9 to 7.4 g/dl), and ultrasonic signs of intraperitoneal fluid. An acute laparotomy showed a small leak from the right corner of the uterus and 2150 ml of blood was removed from the abdominal cavity. One hour before surgery, the patient received a transfusion of one unit of leukocyte‐reduced red blood cell concentrate (RBC), and intraoperatively she received three units of RBCs and four fresh frozen plasma (FFP) units. Near surgery completion, during transfusion of the fourth FFP unit, the patient became hypoxic. Arterial oxygen tension dropped from 100% to 82%, clear frothy sputum emerged from the endotracheal tube, and systemic blood pressure decreased from 140/100 to 90/40 mmHg. The patient received intravenous furosemide (40 + 60 mg), however, there was no response to treatment. Echocardiography within one hour after onset of symptoms revealed normal left ventricular function. TRALI diagnosis was strengthened with a chest radiograph, which showed extensive bilateral lung infiltrates (Figure 1). Computed tomography scan of the chest indicated acute respiratory distress syndrome. Two hours before the first transfusion, the neutrophil count was 12.0 x 10^9^/L (normal range 1.5‐7.5 x 10^9^/L), and it dropped to 0.38 x 10^9^/L at 4 h after onset of symptoms (Figure 2). Simultaneously, the platelet count dropped from 164 x 10^9^/L to 59 x 10^9^/L, and the monocyte count dropped from 0.84 x 10^9^/L to 0.00 x 10^9^/L. The patient was sedated on ventilator support. She received 125 mg methylprednisolonsuccinate intravenously (IV) and following 50 mg of IV hydrocortisone every 6 hours. She had normal body temperature (35.6‐36.9°C) and infection was ruled out, with negative cultures of blood, urine, and endotracheal secretion. The patient's condition improved gradually; after 41 h, she was extubated, but required oxygen support for an additional 60 h. Five weeks later, a chest radiograph showed complete regression of infiltrates and no signs of sustained lung injury.

## 3 SEROLOGICAL ANALYSIS

The patient received blood products originating from eight different blood donors All donors were tested for; anti‐HLA class‐I and anti‐HLA class‐II antibodies, for granulocyte‐ and lymphocyte‐reacting antibodies with a combination of direct and indirect flow cytometric granulocyte/lymphocyte immunofluorescence tests (Flow‐D‐GIFT, Flow‐GIFT, Flow‐LIFT), granulocyte antibodies with a granulocyte agglutination test (GAT) and the LABScreen Multi assay. Antibody specificity was confirmed with monoclonal antibody specific immobilization of granulocyte antigens (MAIGA). Human neutrophil antigen (HNA) typing was performed with a TaqMan real time polymerase chain reaction method (Q‐PCR), as previously described.^6^ We confirmed the findings after 3 months and 6 months. All assays were performed in an International Society of Blood Transfusion (ISBT) granulocyte serology reference laboratory.

## 4 RESULTS

The serum from one blood donor, a 61‐year‐old male, reacted positive against the five donor samples in the flow‐GIFT panel and against patient granulocytes in the direct cross‐match test (Table 1). The antibody showed weak agglutination in the GAT. The LABScreen Multi assay indicated anti‐Fc‐gamma‐receptor‐IIIb. The MAIGA identified an anti‐Fc‐gamma‐receptor‐IIIb (FcGRIIIb; CD16b) antibody. HNA specificity could not be determined further. The Flow‐D‐GIFT was negative. Phenotyping revealed normal CD16b expression on donor granulocytes. Genotyping revealed the donor as FCGR3B*01+,*02+,*03‐. Sequencing the *FCGR3B* gene showed no variant *FCGR3B* alleles. Identical results were found in all assays at 3 and 6 months follow up. Samples were retested in a collaborating ISBT reference laboratory, with identical results.

**TABLE 1:** Blood donor and patient characteristics including results from serological and genetic analysis

Characteristics	Blood donor	Patient
Age in years	61	34
Gender	Male	Female
Previous transfusions	None	None
Pregnancies		3 (one child)
Anti‐HLA class‐I antibodies	Negative	Negative
Anti‐HLA class‐II antibodies	Negative	Negative
Granulocyte cross‐match		Positive with donor serum
Reverse granulocyte cross‐match	Negative with patient serum	
Flow‐GIFT	+ ‐ ++	Negative
Flow‐LIFT	Negative	
Flow‐direct‐GIFT	Negative	Negative
GAT	+	Negative
LABScreen Multi assay	HNA‐1a, −1b, −1c reactive	Negative
MAIGA	Anti‐FcGRIIIb antibody	NA
HNA‐genotyping	FCGR3B*01+,*02+,*03‐, *HNA‐3(ab), HNA‐4(aa), HNA‐5(aa)*	FCGR3B*01‐,*02+,*03‐, *HNA‐3(aa), HNA‐4(ab), HNA‐5(aa)*

## 5 DISCUSSION

This patient sustained TRALI during intensive hemodynamic, echocardiographic, and hematological monitoring. We identified an anti‐FcGRIIIb antibody in a non‐transfused male donor sample. This highly probable trigger made this TRALI case unusual. TRALI is a rare but potentially life‐threatening condition and its diagnosis is primarily based on exclusion. No specific treatment exists and management consist of supportive care. Approximately 80% of patients with TRALI require mechanical ventilation. Steroids offer no benefit, but they are often given, as reported in our case.^7^ Most patients recover without sequelae; however, the syndrome is associated with considerable morbidity; the estimated mortality is 6‐10%. It has been hypothesized that TRALI results from two insults. The first is related to the patient's condition, for example, surgery, sepsis, hematological malignancies, and mechanical ventilation.^8^ These conditions can activate the pulmonary endothelium, prime neutrophils, and cause their subsequent sequestration in the lungs, which lowers the patient's threshold for TRALI. The second insult is the full activation of primed neutrophils by biological response modifiers in the blood product. Our patient displayed a precipitous drop in neutrophils within 4 h after onset of symptoms. This neutropenia was consistent with massive neutrophil agglutination in the pulmonary microcirculation. No other known transfusion reactions are associated with acute transient neutropenia. Our findings support the hypothesis that antibody‐mediated TRALI remains a residual risk associated with transfusions from the non‐transfused male donor population. The donor we identified had no history of allo‐exposure. However, in his occupation as a sailor, he had received repeated vaccines. Previous donor studies have found that approximately 1‐2% of never‐transfused males harbored pan‐reactive or nonspecific leukocyte antibodies.^9^ These naturally occurring antibodies potentially represent autoantibodies, antibodies that cross‐react with bacterial antigens, or antibodies developed after immunizations. In the setting of massive bleeding, current guidelines recommend closely monitoring coagulation with whole blood coagulation methods, like thromboelastography or thromboelastrometry. Implementing those strategies might reduce the use of blood components, and thereby reduce the risk of transfusion reactions.^10^

In summary, this case report illustrates the occurrence of neutrophil‐reactive antibodies in low‐risk donor samples, and the liberal use of blood transfusions. Testing all donors for neutrophil antibodies is expensive and logistically challenging; however, TRALI may represent one of the last major risks in blood transfusion that remains to be minimized with serological testing.

## INFORMED CONSENT

Informed consent to describe this case of TRALI was obtained from donor and patient.

## CONFLICT OF INTEREST

The authors declare no conflicts of interests.

## AUTHOR CONTRIBUTIONS

CNA: Conceptualized the case, had contact with patient and blood donor and wrote the manuscript. TMH: Involved in data analysis and manuscript preparation. ALF: Performed serological analysis and were involved in manuscript preparation. JB: Were involved in data requisition and manuscript preparation. RS: Performed genetic analysis and co‐wrote manuscript. KRN: Were involved in all steps and wrote the manuscript. All authors approved the final manuscript as submitted.

## Data Availability

The authors declare that all data supporting the findings of this study are available within the article.
